# Supplementary material for: Analysis of PPARγ Signaling Activity in Psoriasis
Source: Int J Mol Sci. 2021 Aug 10;22(16):8603. doi: 10.3390/ijms22168603 (PMC8395241; doi:10.3390/ijms22168603)
Supplement: Supplementary file 1 [file ijms-22-08603-s001.zip › Supplemental materials_Analysis of PPARg signaling activity in psoriasis/Pathway models/Models images and html files/PPARG inhibits phenotypes assotiated with psoriasis/134982.html]

nonalcoholic fatty liver


# Disease nonalcoholic fatty liver

|  |  |
| --- | --- |
| URN | urn:agi-disease:non-alcoholic%20fatty%20liver |
| Total Entities | 1 |
| Connectivity | 3418 |
| Name | nonalcoholic fatty liver |

---

|  |  |
| --- | --- |
| ChildConcepts | nonalcoholic steatohepatitis |

---

|  |  |
| --- | --- |
| Pathway | Proteins Involved in non-Alcoholic Fatty Liver Disease |
|  | PPARG inhibits phenotypes assotiated with psoriasis |
|  | Overview. Diseases of the digestive system (chapter 10) |

---

|  |  |
| --- | --- |
| MedScan ID | 9605365 |

---

|  |  |
| --- | --- |
| Alias | non alcoholic hepatosteatosis |
|  | NA-FLDs |
|  | nonalcoholic fatty liver diseases |
|  | non alcoholic fatty livers |
|  | nonalcoholic FLD |
|  | nonalcoholic steatotic liver |
|  | nonalcoholic fatty liver disease |
|  | non alcoholic fatty liver diseases |
|  | non alcoholic hepatic steatosis |
|  | NA-FLD |
|  | non alcoholic FLDs |
|  | NAFLDs |
|  | nonalcoholic liver steatosis |
|  | nonalcoholic fatty liver |
|  | non alcoholic FLD |
|  | non alcoholic liver steatosis |
|  | non alcoholic steatotic livers |
|  | nonalcoholic hepatic steatosis |
|  | non alcoholic fatty liver disease |
|  | nonalcoholic steatotic livers |
|  | nonalcoholic fatty livers |
|  | nonalcoholic FLDs |
|  | non alcoholic steatotic liver |
|  | nonalcoholic hepatosteatosis |
|  | NAFLD |
|  | non alcoholic fatty liver |
|  | non alcoholic steatohepatites |
|  | non alcoholic steatohepatitis |
|  | nonalcoholic steatohepatites |
|  | nonalcoholic steatohepatitis |

---
